# Facially Expressive People are More Popular in Newly Formed Groups: A Social Network Analysis

**DOI:** 10.1007/s10919-026-00514-6

**Published:** 2026-04-27

**Authors:** Alisa Balabanova, Eithne Kavanagh, Tom Kupfer, Bridget Waller

**Affiliations:** Department of Psychology, Nottingham Trent University, Nottingham, UK

**Keywords:** facial expression, social interaction, popularity, social network, facial expressivity, affiliation

## Abstract

Facial expression has been proposed as a key mechanism in forming and maintaining social relationships. Humans have particularly expressive faces, but direct evidence linking individual level expressivity to group-level social bonding is limited. Here we examined whether spontaneous facial expressivity impacts social popularity during initial group formation. Participants (72 groups, *N* = 256) engaged in unstructured online interactions in small groups with strangers during which facial behaviour was quantified using automated analysis. Social popularity was measured by calculating social network indices based on relative liking. More facially expressive individuals were rated as warmer and more cooperative, and occupied more central positions in the group social network. In contrast, facial attractiveness did not predict social standing. These findings support the hypothesis that facial expressivity functions to promote cooperative relationships during group formation.

**Supplementary Information:**

The online version contains supplementary material available at 10.1007/s10919-026-00514-6.

## Introduction

Facial expressions are found in the communication systems of many mammalian species, and their complexity seems to be related to the social complexity of the species, in line with the social complexity hypothesis (Freeberg et al., 2012). As such, facial expression has long been proposed as a key mechanism in the formation and maintenance of social relationships in group-living species (Dobson, 2009; Waller & Micheletta, 2013). In humans, facial expressivity is particularly pronounced, yet direct empirical evidence demonstrating its function in social interaction is lacking. If facial expressivity is linked to social popularity and cohesion, it should be possible to demonstrate this link through analysis of individual differences during social group formation.

Human facial expressions are thought to display internal states (Horstmann, 2003), express views and opinions (Van Kleef et al., 2011), signal response to the actions of others (Ronghe et al., 2017), and/or predict subsequent behaviours (Fridlund & Russell, 2006). The face is attended to more than any other part of the body (Robbins & Coltheart, 2012) and during initial social encounters it produces numerous subtle and complex facial movements (approximately 100 facial movements per minute, (Rollings et al., 2024). People use this facial information to form strong and lasting impressions of one another (Imhoff et al., 2013). For example, individuals are judged as more trustworthy and likable if their expressions are easily and correctly anticipated by others (Chanes et al., 2018; Waller et al., 2017) and more expressive individuals are perceived as more likable by both their interaction partner and unfamiliar others (Kavanagh et al., 2024). These initial impressions can be made very rapidly (Willis & Todorov, 2006), and tend to be long-lasting (Human et al., 2013).

Whether these processes impact an individuals’ ability to integrate within a group and therefore take better advantage of the benefits of group living is unknown. In other primates, there is evidence that inter-individual differences in facial expressivity are related to differential social outcomes at both an individual and group level. More facially expressive dominant male rhesus macaques (*Macaca mulatta*) are more socially connected and have more cohesive social groups. More expressive individuals therefore occupy more beneficial social positions, providing evidence for a social bonding function of facial expression (Whitehouse et al., 2024). When human groups form, competition for social position, attention and resources are a powerful influence on interactional dynamics (Erikson, 2018). Popular individuals have specific advantages, such as wider reach for resources and help from others (Bramoullé et al., 2009), which can relate to lifelong improved health and wellbeing (Hartung et al., 2015). Occupying a central role in a human group is also associated with a number of specific benefits: access to diverse peer support and learning resources (Van Rijsewijk et al., 2018); higher likelihood of securing leadership positions (Burt, 2005); stronger sense of social identification (Graupensperger et al., 2020) receiving more citizenship behaviours and fewer counterproductive behaviours in work settings (Malamut et al., 2021); lower levels of loneliness (Malamut et al., 2021) and anxiety (Long et al., 2020). If facial expression can be used to leverage social popularity within groups, this is therefore likely to incur fitness benefits.

The mechanism by which facial expression has been suggested to act as a social bonding mechanism is by providing other individuals with information about what is likely to happen next, as signals of motivation or intention (Fridlund, 2017; Waller et al., 2017, 2025). Social partners who are more predictable tend to be preferred, as predictability is linked to heightened perception of control over the interaction (Bolt & Loehr, 2017). Predictability therefore offers an opportunity for others to react with an appropriate response and avoid conflict (Waller et al., 2017), regardless of whether the expression can be categorised with negative or positive valence. Similarly, facial expressions that fit the social context and are anticipated correctly are associated with greater likability (Chanes et al., 2018), pointing to the role of expressivity in honest, cooperative signalling (Reed et al., 2012). Facial behaviour has also been linked to other characteristics that are important during group formation, such as warmth and competence (Abele et al., 2008; Fiske et al., 2007; Ponsi et al., 2016). These characteristics are perceived as informative of the intentions of others and form diagnostic impressions of both individual and group-level interactions (Cuddy et al., 2008). Warmth has been seen of particular importance in relation to group membership as it may be indicative of other’s cooperation intentions (Eisenbruch & Krasnow, 2022). Facial expressivity has also been used to inform perceptions of trustworthiness and cooperation (Stouten & De Cremer, 2010) as well as influencing trust and cooperation (Campellone & Kring, 2013), which is also of potential importance in group-based scenarios (Shinada et al., 2010).

To date, the measurement of facial expressivity has relied almost exclusively on self-report (Fultz et al., 2024), or through the judgement of others (Stagg et al., 2014). Analysis has also focussed on the emotional states assumed to be associated with the facial behaviour, rather than objective quantifiable data about the facial behaviour per se (Crivelli & Fridlund, 2018). However, self-and-other-based perception measures may not reflect actual behaviours (Breil et al., 2022), and facial expressions do not always reflect internal states (Barrett et al. 2019a). The Facial Action Coding System (FACS, Ekman & Friesen, 1978) can be used to measure individual muscle movements as they appear in each time-frame of a recorded interaction, and as such is a valuable tool in quantifying real-world facial behaviour. Until recently, however, manual coding of real-world social interactions has been too time consuming for meaningful analysis. Now, automated systems are available that allow for analysis of much larger datasets. For example, both Kavanagh et al. (2024) and Rollings et al. (2024) utilise automated coding methods to show that people who produce more facial expressions are liked more by their interaction partners in online and in-person dyadic settings.

Historically, research on facial expression and nonverbal communication has relied heavily on static images as experimental stimuli, a pattern documented across general face-processing research (Dawel et al., 2021), studies of emotional expression (Barrett et al., 2019), and work in social perception more broadly (Satchell et al., 2023). This emphasis on static, decontextualised displays has provided valuable theoretical insight but offers only a limited approximation of how expressive behaviour unfolds in real social interaction. By contrast, the current study uses dynamic facial expressivity captured during real-time group interactions, allowing us to examine nonverbal behaviour with greater ecological validity. This methodological approach directly addresses the field’s longstanding reliance on static imagery and represents a significant step toward understanding how expressive signals function in naturalistic social contexts.

Here, we tested whether individual differences in facial expressivity are associated with social popularity during the formation of human social groups. We quantified facial expressivity during the unscripted social interaction of groups of strangers. We then used social network analysis to measure popularity of individuals within groups based on relative liking across group members. The social network approach allows us to take into account how people are perceived, how they perceive others and what their position is in comparison to other individuals within the group (Zhang & Luo, 2017) as a measure of social popularity. We also controlled for additional factors which are known to impact interpersonal perception, such as the ‘halo’ effect of attractiveness (Batres & Shiramizu, 2023) and time spent talking (Hirschi et al., 2023). We predicted that displaying higher overall expressivity would be positively associated with more favourable perceptions from others (in terms of likability, warmth, trustworthiness, cooperation, trust behaviours and behavioural intentions) and with occupying a more central position within the group (i.e. social popularity).

## Method

### Phase 1: Social Interaction

#### Participants

In total, 262 participants were recruited and randomly assigned to groups of four or three (72 groups) via an online recruitment platform (Prolific; http://www.prolific.co). During the process of the study, 8 subjects were excluded from the final data analysis for various reasons: one person participated twice, so their second participation was disregarded; two people were excluded due to missing questionnaire data; one person dropped out during the initial video call; and four people’s video data was not reliably coded by the software iMotions due to inability to detect facial landmarks. Thus, the final sample consisted of 254 participants aged 25–35 (M_age_ = 30.2, SD_age_ = 3.12), 121 of which identified as female, and 133–as male. We restricted group size to maximum four as this provides the optimal environment for individuals to actively partake in a single conversation (Dunbar, 1995; Henzi et al., 2007). All participants were fluent English speakers and resided in the UK. The sample size was informed by a G*Power calculation at an alpha of 0.05, sufficient to detect a small, standardised effect size of g = 0.1 with 0.95 power for each outcome variable. All subjects gave informed consent and were compensated with £5 for their participation.

**Fig. 1:**
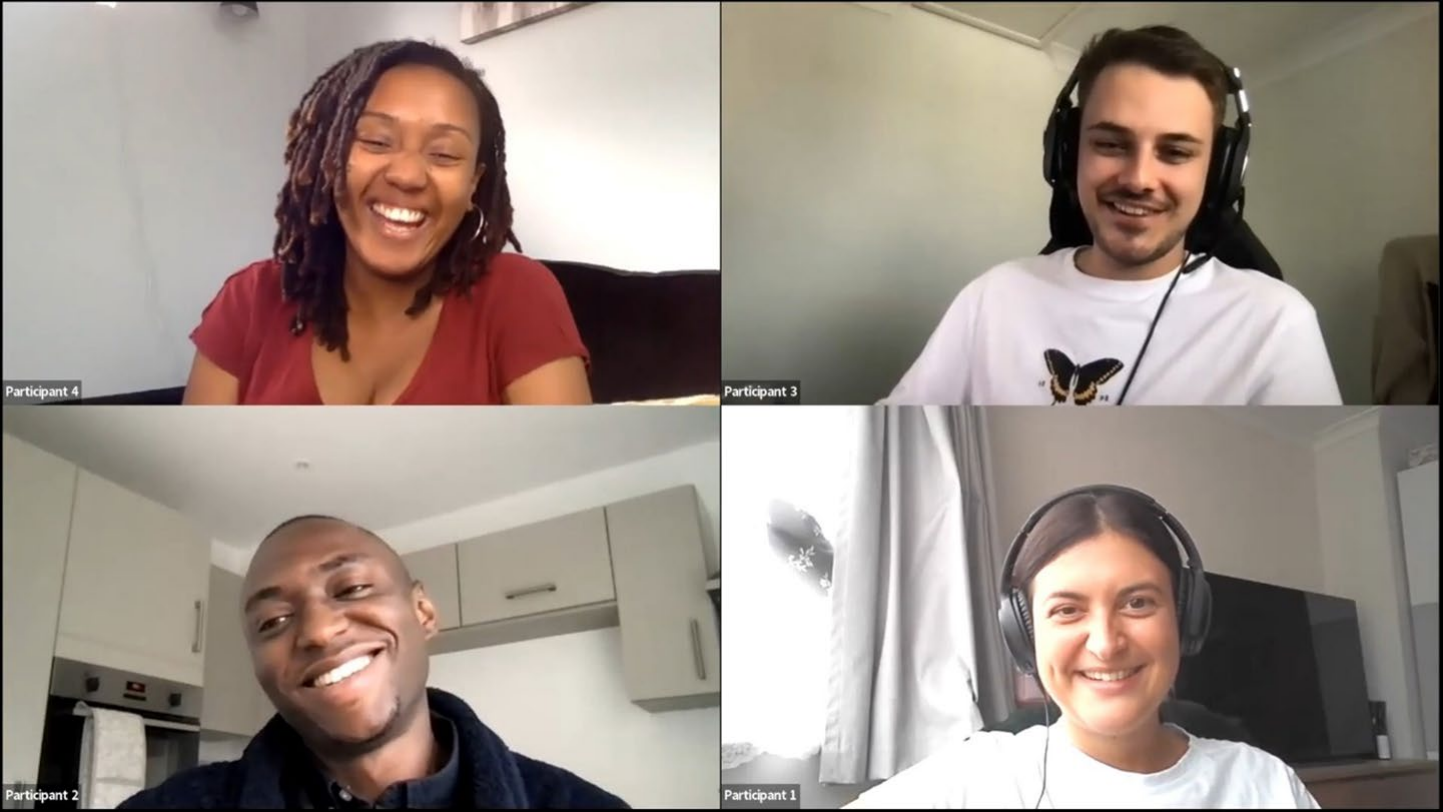
An example four-person group (Group 25) engaged in 5 min of spontaneous social interaction (facial expressivity measure extracted using FACS)

#### Procedure

Participants joined a video call (via Zoom software; https://zoom.us/) in groups of four or three people (see Fig. 1.). Prior to joining the call, subjects were instructed to ensure adequate lighting was used and their faces were illuminated well (to ease facial expression coding); they were asked to sit in front of a neutral background, and, if possible, wear neutral clothes. Once participants joined the call, they entered a waiting room, in which they were not able to see the other participants. The experimenter conducted audio and video checks with each individual separately, and then welcomed all participants to the call at once. Participants were then asked to keep their videos on during the interactive parts of the study (i.e., social interaction) and consented to the session being recorded by the experimenter. Individuals first gave a short greeting to the other participants (always in the order of participant number) and were then asked to engage in short (up to five minutes) spontaneous interaction without the experimenter present. The only instructions participants received were ‘*You can talk about anything that you like between each other. For example*,* you can talk about your experiences with Prolific*,* or any other topic you would like*.’ Following the social interaction, the experimenter re-entered the conversation, instructed the participants to switch off their cameras/microphones and guided the participants through the Trust Game via the in-built Zoom poll option. Instructions were displayed on the screen and explained verbally by the experimenter. Participants first played the cooperative condition (common pot), followed by the individual condition. Participants only engaged in one round of the game and were not made aware of the game’s outcomes or the other players’ responses. Finally, participants were asked to complete a questionnaire, which measured participants’ perceptions of each other. Subjects were asked to remain on the call while completing the questionnaire but were instructed to mute their microphones and turn their cameras off, so as not to interfere with each other.

**Fig. 2:**
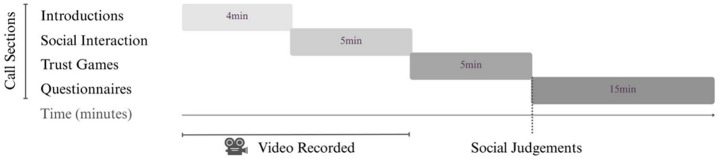
Timeline of the video call procedure

#### Measures

##### Facial Expressivity

A FE score was calculated based on the video data collected during the group’s social interaction. Specifically, we coded all occurrence facial behaviour during the unscripted interactions participants engaged in within their groups when the experimenter was not present, which lasted for approximately 5 min. We used the Facial Action Coding System (FACS) to quantify all visible muscle movements in the face, referred to as Action Units (AUs) using automated iMotions software that utilises Emotient’s FACET technology to extract 16 distinct AUs: 1, 2, 4, 5, 6, 7, 9, 10, 12, 14, 15, 17, 18, 20, 24 and 28 (iMotions Biometric Research Platform, SW Version, 2001). We excluded AU25, AU26 and AU43 as they are closely associated with speaking and blinking, as well as ‘Smirks’ as they are already coded as an asymmetrical AU12. The automated facial movement coding has shown high consistency with expert human coders (Benitez-Quiroz et al., 2016). From the resulting data we calculated six facial expressivity measures for each participant: duration, diversity, rate, repertoire, combination repertoire, and corrected repertoire (see Table 1). Following data-driven approaches from Kavanagh et al. (2024) and Rollings et al. (2024), we conducted a principal components analysis (PCA) in R (R Core Team, 2021), using the ‘*psych*’ package. The analysis revealed that all six measures loaded moderately high on a single component. Therefore, to form an overall facial expressivity score, we calculated the average of the transformed Z-score distributions of each FE measure. This approach allows us to consider facial movement without segregating based on valence or assumed underlying emotional state.

**Table 1:** FACS Measures used to produce an overall expressivity score

Measure	Calculation
Duration	The percentage of frames in which each AU was produced, summed per participant.
Diversity	The number of unique AUs and how they are represented. This measure combines Repertoire and Duration to show how evenly facial expressions are displayed. Calculated based on the formula from Scheider et al. (2014).
Rate	The number of AUs produced per minute.
Repertoire	The total number of unique AUs produced in the first 20 s.
Corrected repertoire	The total number of unique AUs produced out of the first 25 AUs produced.
Combination repertoire	The total number of unique AU combinations (i.e. simultaneous productions of 2 or more AUs) in the first 20 s

##### Person Perception Scores

To capture how participants perceived other members of their group, we utilised various measures: social popularity, warmth, competence, trust, cooperation perceptions, and behavioural intentions. The following (listed below) were all measured using a scale slider ranging from 0 ( = ‘not at all’) to 100 ( = ‘extremely’) unless stated otherwise.

##### Social Popularitpularity

We first measured the degree to which each participant was liked by their interaction partners with a single-item asking, “*How much do you personally like X?”*. The given values were then used to compose directional social networks of each group, from which we extracted degree centrality, as our measure of social popularity. Degree centrality reflects how well-connected an individual is within their social network, taking into account the strength of each connection they hold with the other group members as a composite of their out-degree (the value they have given to each person individually), their in-degree (the value they have received from each other person individually) and the links between the rest of the group members (Zhang & Luo, 2017). We further standardised the resulting degree centrality scores in accordance to group size (N = 3 vs. N = 4) by dividing the obtained raw score by the maximum score possible (400 for groups of 3 and 600 for groups of 4) in order to control for the fact that larger groups would artificially increase centrality measures. This allowed is to compare scores obtained in groups of 3 and groups of 4 on the same scale. Thus, resulting in a single value of social popularity for each participant ranging between 0 and 1 with lower scores indicating lower social popularity, see exact formula below. We refer to social popularity as ‘network degree centrality’ in the results section.

$$\:\:Standardised\:{Degree\:Centrality}_{i}=\frac{{In\:Degree}_{i}+\:{Out\:Degree}_{i}}{2\:x\:\left(n-1\right)\:x\:100}\:$$

Where: n = size of the individual’s group, In-degree = sum of liking received, Out-degree = sum of liking given.

##### Warmth and Competence

We measured perceptions of warmth and competence with a six-item questionnaire (three items per characteristic: *friendly*,* positive*,* likable*–for warmth; *able*,* capable*,* competent*–for competence) adapted from Williams and Bartlett (2015). For example, participants were asked to report to what extend the words ‘*friendly’* (for warmth) and ‘*able’* (for competence) described the other participant(s). The obtained scores were averaged per participant resulting in one overall score for warmth, and one for competence. Internal consistency was excellent for both scales (α_warmth_ = 0.95, α_competence_ = 0.97).

##### Behavioural Intentions

To capture future behavioural intentions, a one-item measure was used asking each subject to rate each of the other participants in their group: ‘*how likely are you to engage in further conversation with X outside of the study’*.

##### Trust and Cooperation Perceptions

Perceptions of trustworthiness and cooperations were measured with single-item measures, asking ‘*In your opinion*,* how trustworthy/cooperative are the people you interacted with in this study?*’. Each participant gave a single measure of trustworthiness, and a single measure of cooperativeness for each individual group member.

##### Trust and Cooperation Behaviours

Behaviours were measured with an adaptation of the Public-Goods Game based on Berg at al. (1995). In this game subjects were given 10 imaginary tokens each and asked to choose how many of those tokens they wanted to contribute to a (1) common pot (cooperation), and (2) give to the other participants (trust). The amount of the given coins was then multiplied by 3 and was (1) shared equally between the participants, (2) given to the other person to divide between themselves and the initial player. The amount contributed to the pot was used as a proxy for measuring cooperativeness; the amount given to other participants was used as a proxy for trusting behaviours; the amount returned back was used as a measure of trustworthiness; and the amount received from others was used as a proxy of how trusted participants were by others. Each subject played each iteration of the game (both versions 1 and 2) only once and was not informed of the outcome of the game in terms of how many tokens they received back. For example, in the first iteration participants were asked “*How many coins would you like to donate to the common pot of gold?*”, while in the individual iterations participants were asked, e.g. “*How many coins would you like to put in Participant X’s pot?*”. Answers to these questions were given on a 11-point Likert scale between 0 and 10. To determine the division of the outcome, the question was worded as “*What percentage of the multiplied coins do you want to return to Participant X?*”, answers given on a 11-point Likert scale between 0% and 100%. Participants were not informed what amount of the coins others returned to them.

### Phase 2: Third-Party perceptions

#### Participants

In total, 620 independent raters between the ages of 18 and 80 (M_age_= 40.14, SD_age_ = 12.44) were recruited via Prolific to rate still images of the original participants. Our target sample size was informed by prior research (Hehman et al., 2018) and an estimation for stable ratings based on previously collected data, suggesting that a stimulus must be rated by 20 individuals to reach a representative score stable at the population level. The collected ratings were aggregated over stimulus for the purpose of our analyses, with each rating representing the average score of this stimulus as given by the independent raters. All raters were fluent English speakers and resided in the UK at the time of data collection. The sample consisted of 311 raters identifying as ‘female’, 307 identifying as ‘male’ and 2 who preferred not to disclose their gender. Subjects gave informed consent and were compensated with £2 for their participation.

**Fig. 3:**
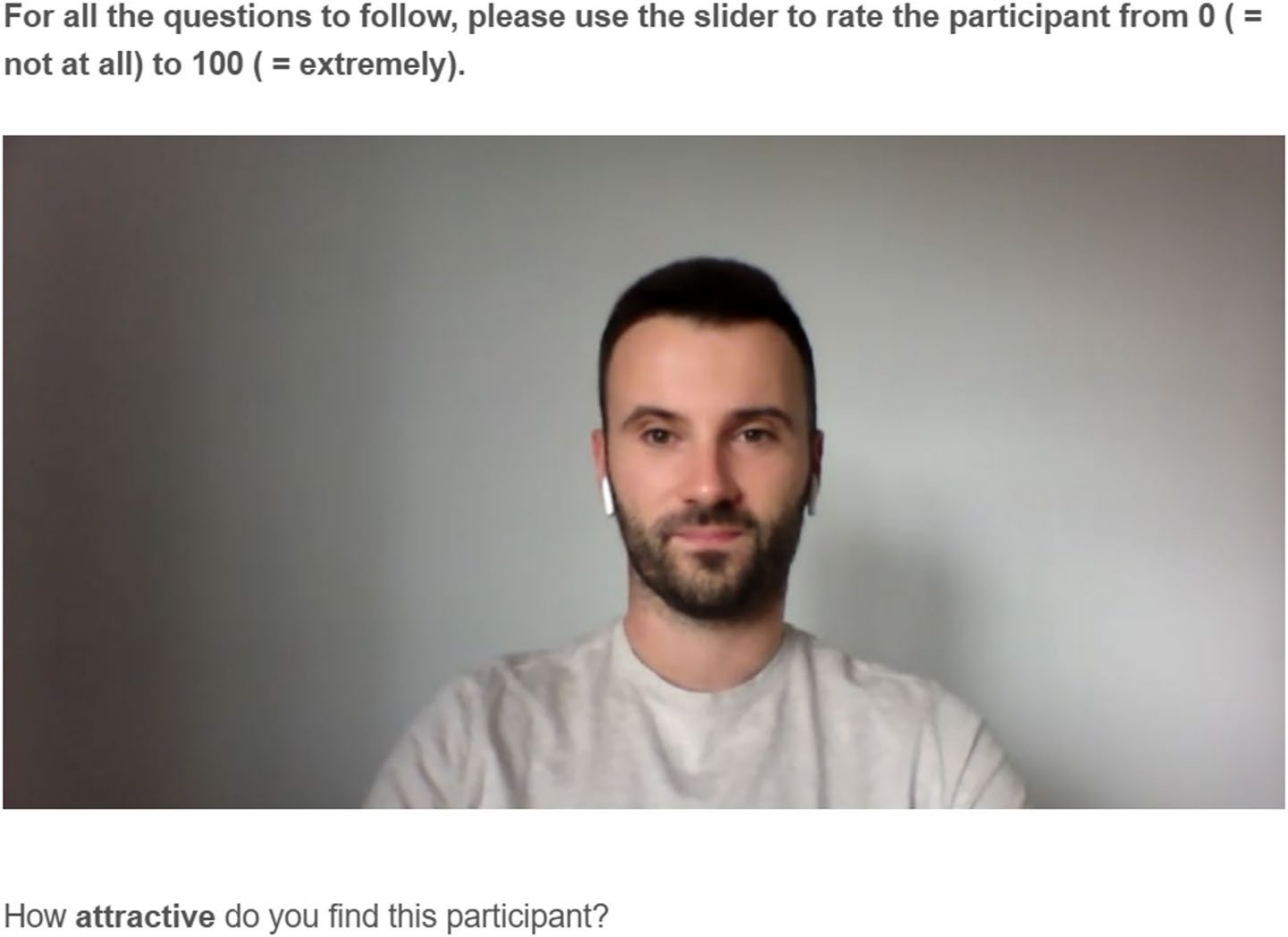
An example still image taken for the independent ratings

#### Procedure

Following the initial data collection, independent raters engaged in a short online survey in which they were asked to rate still images of the participants on attractiveness (as well as the same person-perception characteristics that the participants filled in during the video call). Each rater rated 4 randomly-selected images in a counter-balanced order (see Fig. 3 ).

#### Measures

##### Stimuli

Still photographs of the original participants were taken from the video during audio checks performed by the experimenter (prior to participants being admitted to the video call interaction and meeting the rest of the participants). These photographs were taken when no other participants were present and images were selected to represent the participants with still neutral faces (with no facial movement being detected on the face) in good lighting conditions (see Fig. 3). We used still images to control for the effect of facial movement on person-perceptions (Golle et al., 2014).

##### Attractiveness

 Attractiveness was measured with a single item, asking independent raters ‘*How attractive do you find this person?’* Inter-rater agreement for this item was assessed using a random-effects intraclass correlation coefficient (ICC) and showed excellent reliability (ICC = 0.84).

##### Other Person Perception Measures

Naïve observers were also asked to rate the participants on liking, behavioural intentions, warmth, competence, trustworthiness and cooperation perceptions using the same items outlined above.

#### Overall Statistical Analysis

All data processing and analyses were conducted using R statistical software (Version 4.1.1; R Core Team, 2021). Linear mixed models were constructed with the *lmer* function from the *lmerTest* package and all included Expressivity, Perceived Attractiveness and Group Size as fixed effects, and the Group Number as a random effect. Eleven models were conducted altogether–one for each outcome variable measured (liking, warmth perception, competence perception, cooperativeness perception, trustworthiness perception, behavioural intention, trust game outcome–trusting, trustworthy, trusted and cooperative, and degree centrality). Each outcome variable was modelled with the following structure:

$$ \begin{aligned} Outcome_{{ij}} = & \beta _{0} + \beta _{1} Expressivity_{{ij}} + \beta _{2} Perceive\:Attractiveness \\ & \quad + \beta _{3} Group\:Size + u_{{\left\{ {0j} \right\}}} + \in _{{\left\{ {ij} \right\}}} \\ \end{aligned} $$

The model on degree centrality differed in that group size was not included as a random effect as degree centrality scores were standardised according to group size. Effect size estimates were calculated with the *partR2* function from the *partR2* package. Figures were built with the use of the *ggplot2* package. The *igraph* package was used to construct weighted matrices based on the liking scores participants gave to each other, see Fig. 5  for example. Degree centrality for each individual was extracted from the network and modelled as a function of individuals’ facial expressivity during the unscripted social interaction.

##### Control Variables

**Fig. 4:**
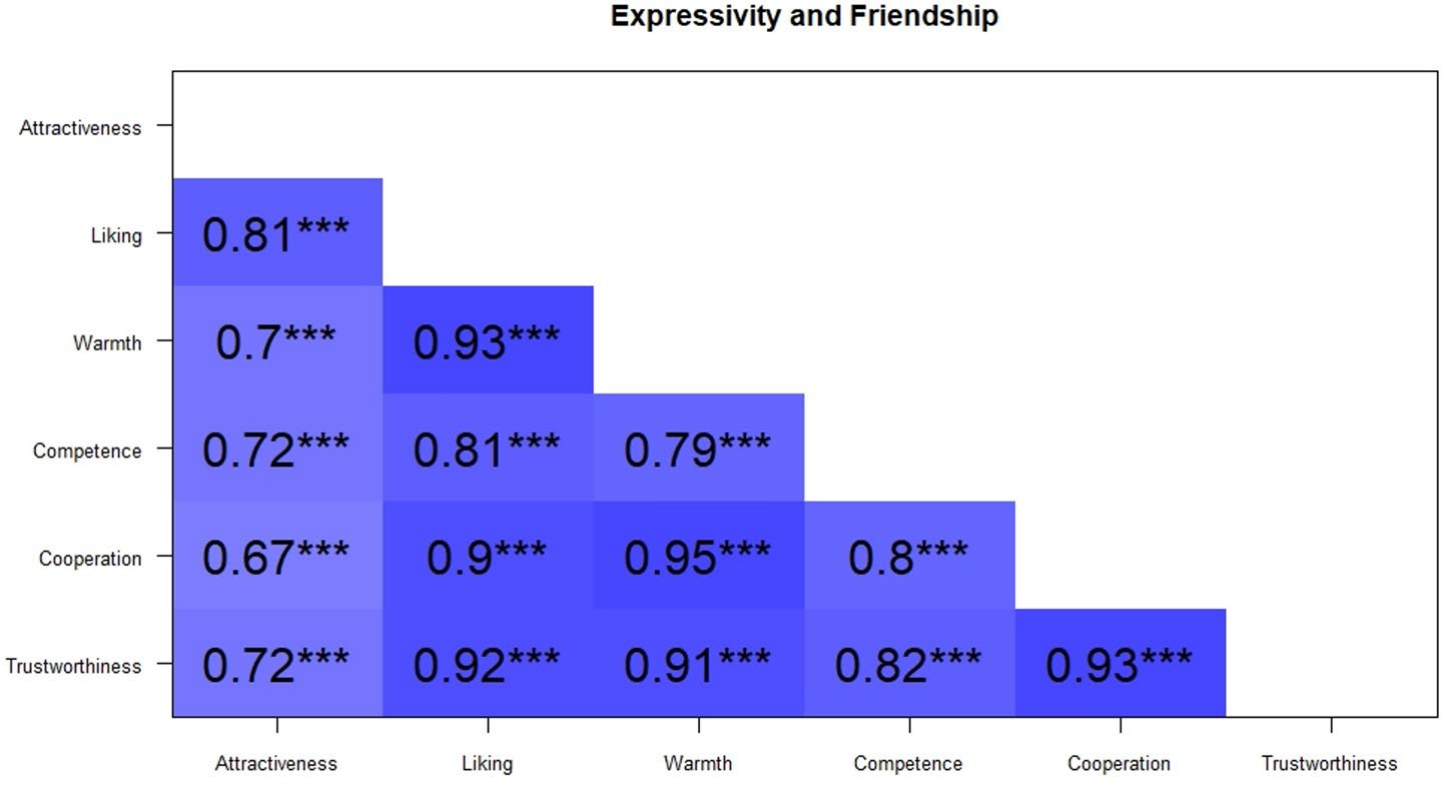
Correlations between third-party perceptions

To test whether the ‘halo’ effect of attractiveness was present in our sample, we used the *corr.test* function from the *psych* package to model the relationships between all person-perception variables, and we visualised those relationships using the *corPlot* function. All variables correlated significantly and highly with each other (see Fig. 4.). Therefore, we included only attractiveness as a predictor in our models (controlling for the halo effect of attractiveness). Full models with all control variables can be found in the Supplementary File (no variable was found to be a significant predictor).

## Results

### Expressivity and Person Perception during Group Formation

We first modelled the relationship between facial expressivity and person-perception characteristics. As seen in Table 2., more expressive individuals received higher ratings for individual liking, and were perceived as warmer and as more cooperative by their group members. Participants did not display higher behavioural intentions towards more expressive individuals and did not perceive more expressive individuals as more competent or trustworthy. Additionally, attractiveness of the participants predicted only warmth perceptions (although to a lesser degree than FE) and trustworthiness perceptions, such that more attractive individuals were perceived as warmer and more trustworthy. The models’ random effects components are reported in Table 3. below.

**Table 2:** Effects of expressivity and attractiveness on person perception scores

Outcome	Predictors	β	CI	t	df	*p*	*R*²sp
Liking	Expressivity	2.63	[0.57, 4.74]	2.49	215.5	0.013	0.02
	Attractiveness	0.11	[− 0.02, 0.23]	1.66	220.19	0.099	0.01
	Group size	− 3.61	[− 8.68, 1.45]	− 1.39	70.79	0.168	0.01
Warmth perception	Expressivity	3.76	[1.79, 5.74]	3.72	229.31	< 0.001	0.04
	Attractiveness	0.15	[0.03, 0.28]	2.52	234.85	0.012	0.02
	Group size	− 3.25	[− 7.29, 0.78]	− 1.57	71.19	0.12	0.01
Competence perception	Expressivity	1.52	[− 0.35, 3.42]	1.58	227.06	0.11	0.01
	Attractiveness	0.10	[− 0.01, 0.22]	1.75	232.49	0.08	0.01
	Group size	− 3.98	[− 7.96, − 0.01]	− 1.95	71.77	0.054	0.02
Cooperativeness perception	Expressivity	2.81	[0.74, 4.89]	2.65	227.38	< 0.01	0.02
	Attractiveness	0.12	[− 0.01, 0.25]	1.87	232.90	0.062	0.01
	Group size	− 5.43	[− 9.77, − 1.09]	− 2.45	71	0.017	0.04
Trustworthiness perception	Expressivity	1.19	[− 1.37, 3.78]	0.91	226.33	0.366	0.003
	Attractiveness	0.21	[0.06, 0.37]	2.65	231.79	< 0.01	0.02
	Group size	0.17	[− 5.28, 5.61]	0.06	71.18	0.949	0.000
Behavioural intention	Expressivity	1.66	[− 1.63, 4.98]	0.98	208.09	0.325	0.002
	Attractiveness	0.11	[− 0.09, 0.32]	1.12	212.06	0.263	0.003
	Group size	1.61	[− 7.43, 10.62]	0.35	69.95	0.73	0.001

Each outcome is tested in a separate model. β, fixed-effect estimate; CI, confidence interval; R^2^sp, semi-partial sum of squares. All models include a random intercept for Group

**Table 3:** Random effect of group across the person perception models

Outcome	Intercept variance	SD	Residual variance	Residual SD	ICC
Liking	78.77	8.88	102.35	10.12	0.42
Warmth perception	39.82	6.31	98.04	9.9	0.29
Competence perception	40.55	6.37	88.51	9.41	0.31
Cooperativeness perception	47.75	6.91	107.43	10.36	0.31
Trustworthiness perception	76.97	8.78	164.09	12.81	0.32
Behavioural intention	272.9	16.52	253.9	15.94	0.52

ICC, intraclass correlation coefficient, computed as τ^2^/ (τ^2^ + σ^2^). Variance components reflect random intercepts for group

The outcomes of the Trust Game were also modelled, revealing that more expressive individuals acted in a more trusting manner towards their team members. However, they did not receive more trust by the respective counterplayers. Attractiveness did not seem to play a role in any of the behavioural outcomes associated with the trust games. See Table 4. for full results.

**Table 4:** Effects of expressivity, attractiveness, and group size on trust behaviours measured via the trust game

Outcome	Predictors	β	SE	t	df	*p*	*R*²sp
Trusting	Expressivity	0.66	0.29	2.29	247.76	0.022	0.02
	Attractiveness	0.01	0.02	0.30	249.85	0.767	0.000
	Group size	− 0.30	0.47	− 0.63	78.09	0.528	0.002
Trusted	Expressivity	0.04	0.14	0.27	198.43	0.784	0.000
	Attractiveness	0.02	0.01	1.93	201.09	0.055	0.007
	Group size	− 0.31	0.48	− 0.65	70.14	0.518	0.004
Trustworthy	Expressivity	1.89	1.72	1.09	249.70	0.274	0.005
	Attractiveness	− 0.13	0.10	− 1.25	246.84	0.214	0.006
	Group size	− 3.08	2.57	− 1.20	92.29	0.234	0.006
Cooperative	Expressivity	− 0.17	0.27	− 0.62	249.91	0.534	0.001
	Attractiveness	0.01	0.02	0.35	249.14	0.728	0.000
	Group size	0.00	0.42	0.01	82.23	0.995	0.000

### Expressivity and Social Popularity

**Fig. 5:**
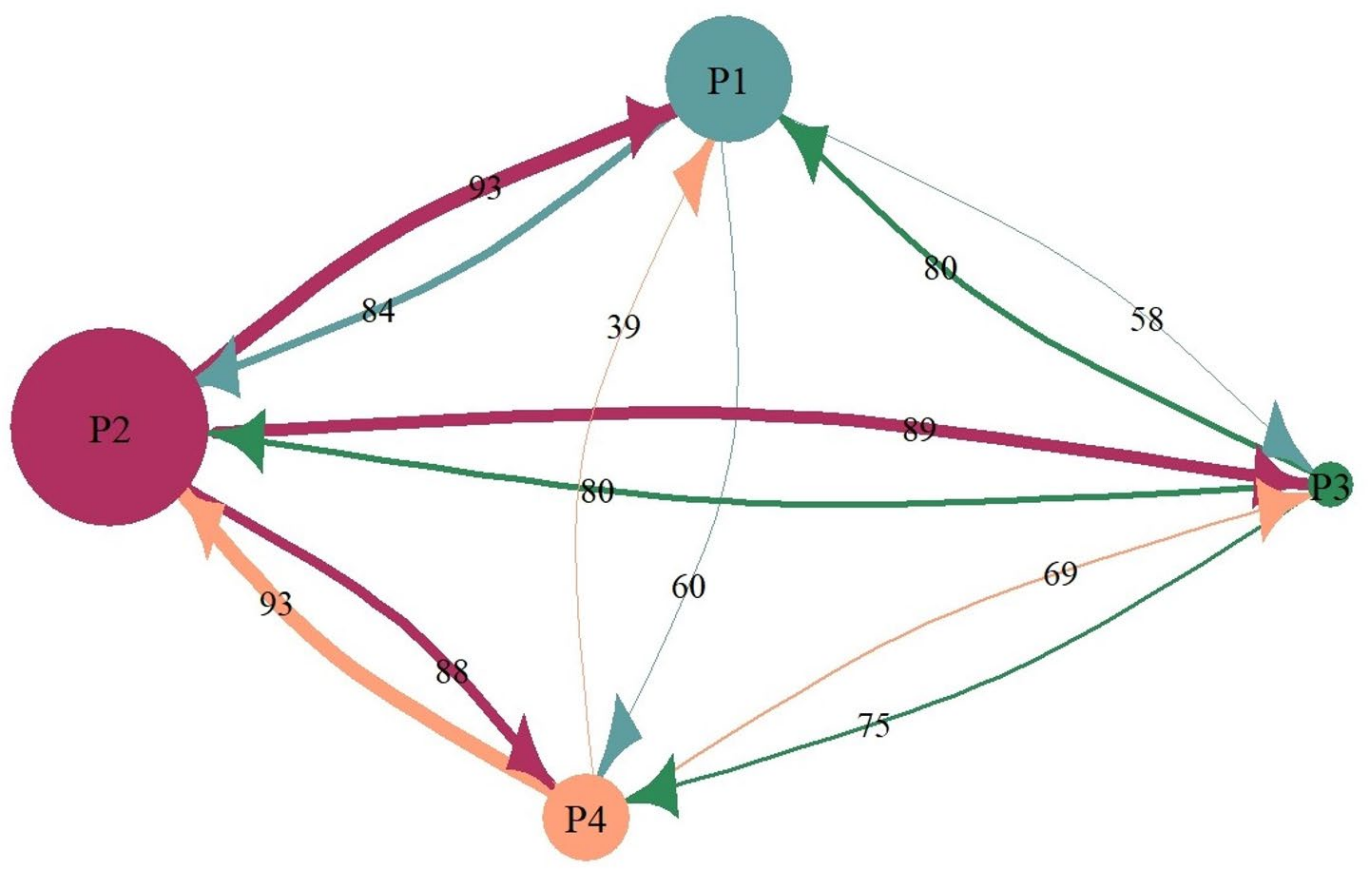
An example of a group’s network structure based on liking scores between individuals (Group 30). Nodes represent participant numbers, edges display liking scores, with thicker edges suggesting higher scores. The size of the nodes is proportional to the individuals’ facial expressivity, and the position in relation to other nodes reflect the reciprocal relationship between the group members–nodes located closer together suggest higher reciprocal liking

To estimate how popular and well-integrated a participant was in relation to their group members, we calculated group-level social network attributes. Particularly, we focussed on degree centrality as a measure of individual popularity within the group setting. See Fig. 5. for an example of the created social network structure.

**Fig. 6:**
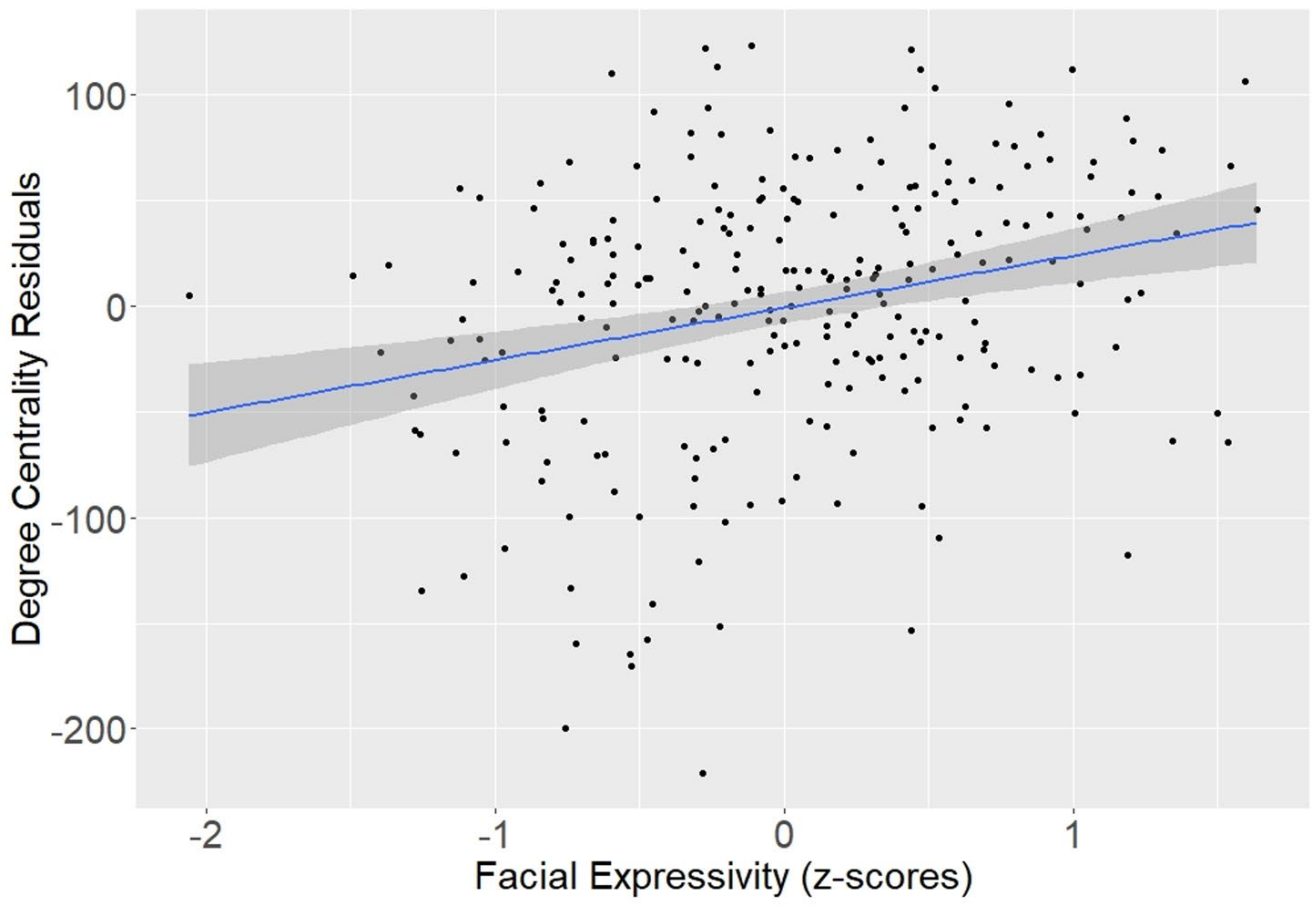
Relationship between facial expressivity and network degree centrality

Our findings suggest that people who display higher levels of facial expressivity during the social interaction, occupy more central positions in the social network of their group, as indicated by higher degree centrality scores (see Fig. 6.). The figure depicts the degree centrality residuals transformed as a function of group size (as degree centrality is affected by group size) to show the overall effect of facial expressivity on degree centrality, our measure of group popularity.

A mixed effects regression analysis was conducted to predict the degree centrality of individuals based on facial expressivity and perceived attractiveness (fixed effects), with group included as a random intercept. The model revealed that facial expressivity significantly predicted degree centrality, β = 0.025, 95% CI [0.01, 0.04], t (193.3) = 3.93, *p *< 0.001, accounting for 2.2% of unique variance (semi-partial *R*^2^ = 0.022). Individuals with higher FE were found to occupy more central positions in their social group. Perceived attractiveness was not found to be a significant predictor of how central individuals were in their social group, β = 0.001, 95% CI [− 0.001, 0.001], t (195.3) = 0.98, *p* = .34, *R*^2^*sp* = 0.001. The model included a random intercept for Group, which showed a variance of 0.01 (SD = 0.09), indicating notable between-group variation in standardized degree centrality. The residual variance was 0.003 (SD = 0.06). The intraclass correlation coefficient (ICC) was 0.74, suggesting that 74% of the variance in degree centrality occurred between groups rather than within groups.

To evaluate whether including Group as a random intercept improved model fit, we compared the model with the random effect to a model without it using a likelihood ratio test. The model including the random intercept fit significantly better than the model without it, χ²(1) = 154.69, *p* < 0.001, indicating that accounting for between-group variability substantially improved the model’s explanatory power.

### Additional Controls

Two additional variables were measured and then controlled for in our model to account for possible confounding effects: smiling and time talking. Smiling was measured by calculating the duration of AU12 per participant during the unscripted interaction. Time talking was measured by calculating the percentage of time each participant spent talking during the unscripted interaction. Both measures were included as control variables into our model of popularity. Results indicated that while smiling did significantly predict degree centrality this was not as strong a predictor as overall expressivity, and time talking did not have an influence on popularity within the group. See Table 5. for full results.

**Table 5:** Effects of expressivity, attractiveness, smiling, time talking and group size on degree centrality estimates

Predictors	β	CI	t	Df	*p*	*R*²sp
Expressivity	0.019	[0.01, 0.03]	2.73	191.3	0.007	0.01
Attractiveness	0.0001	[0.00, 0.00]	0.27	191.4	0.786	0.000
Smiling	0.001	[0.00, 0.00]	2.79	206.7	0.006	0.02
Time talking	0.024	[− 0.03, 0.08]	0.81	184.3	0.430	0.000

## Discussion

Here we tested whether individual differences in facial expressivity are associated with social popularity during group social interactions. We found that more expressive people occupied more central social network positions (based on relative liking among group members). We also found that more facially expressive people were more well-liked by other group members, perceived as warmer and more cooperative, and acted in a more trusting manner during economic games. In contrast, facial attractiveness positively predicted perceptions of warmth, but not of relative liking and cooperativeness, and did not impact social popularity (social network position) within the group. Together these findings suggest that facial expressivity plays a prominent role in forming social bonds within human social groups. While it is possible that facial expressivity increased in response to better social popularity within the group, facial expressivity has been shown to be a relatively stable individual trait (Kavanagh et al., 2024), and not a highly variable behaviour. Thus, we discuss the observed association between FE and social popularity in line with theoretical ideas that suggest that interpersonal behaviours can impact group structure and formation.

Humans live in unusually large and complex social groups and several cognitive and behavioural strategies have been proposed as adaptive solutions to maintaining cohesion in these groups (Dunbar, 2024). Facial expression has been proposed as a potential solution (Waller et al., 2025), and our findings support this hypothesis. Facially expressive individuals are perceived more favourably both by their interaction companions and by naïve observers (Kavanagh et al., 2024; Rollings et al., 2024), but our results build on this by showing that facial expressivity can positively influence the role participants take within a group as early as the first interaction. Popularity during the initial group formation process is of particular importance to stabilising one’s social position as the group develops (Sewell & Chen, 2015) and can have long-lasting effects on how a person attracts social contacts over time. For example, it is reported that popular individuals are perceived as more desirable friends (Bravo et al., 2022) and have more diverse real-world social networks (Bramoullé et al., 2009), which is associated with heightened well-being and social cohesion (Ramos et al., 2024). Popularity and centrality within a group is also related to wider access to resources (Bramoullé et al., 2009), more help from in-group members (Scott & Judge, 2009), and higher growth and status opportunities (Burt, 2005). Group-level measures can also impact dyadic relationships beyond the group context. For example, popular individuals tend to have higher influence in subsequent dyadic interactions, attracting more compliance from others (Lansu & Cillessen, 2015). Considering these important benefits that group centrality and popularity can offer the individual and their association with facial expressivity, in line with evolutionary perspective, facial behaviour could have been selected as a mechanism for aiding group integration and maintaining cohesion.

Facial expressivity also predicted higher perceptions of warmth and cooperativeness, but not competence. Perceptions of warmth are thought to be important in relation to group membership as indicative of other’s cooperation intentions (Eisenbruch & Krasnow, 2022). If facial expressions are predictive of potential action (Crivelli & Fridlund, 2018; Fridlund, 2017; Fridlund & Russell, 2006; Waller et al., 2017), and not solely read-outs of emotion, it could be that more expressive people are seen as more predictable and therefore better potential cooperative partners. However, during our economic games, while more facially expressive participants were more trusting towards other group members, they were not more trusted by others, nor did they behave more cooperatively towards others. Previous research suggests that trusting behaviours can be impacted by the facial expressions of the receiver of the action, at least in experimental settings. For example, Campellone and Kring (2013) show that displays of anger influenced trusting decisions during initial encounters, while Centorrino et al. ( 2015) found that genuine smiles positively predicted willingness of partners to trust others. Our findings suggest that in naturalistic social interactions facial expressivity is associated with exerting more trust but not receiving more trust from others. Prior work shows that facial expressivity is loosely tied to temperament and prosocial dispositions such as agreeableness (e.g., Kavanagh et al., 2024), which may explain the association between expressivity and higher trust in others, as more agreeable individuals have been shown to act more trustingly (McCarthy et al., 2017). However, observers may not necessarily infer trustworthiness from facial expressivity and can rely more on behavioural consistency or other contextual information, therefore not reciprocating the received trust.

Facial attractiveness has been suggested to play an influential role in person-perception and group structure, generally affording a social advantage as a ‘halo effect’ (Jæger, 2019). Here, however, we found no relationship between attractiveness and individual liking or social popularity within the group. Attractiveness has been well established as an important factor in dyadic contexts (Little et al., 2011) but our results suggest that its relevance in a group context is reduced. In dyadic settings, facial attractiveness of the people interacting can positively influence enjoyment from the interaction (Sprecher & Hatfield, 2022). However, in group settings, likability, openness and prosocial behaviours emerge as more important factors impacting popularity (Lansu et al., 2023). Hogg and Turner (1985) suggest that adherence to group membership is of higher importance than individual appeal during group formation. It is, therefore, possible that facial expressivity is indicative of behaviours that are particularly relevant in a group setting, such as cooperativeness. For instance, facial expressivity has been shown to improve conflict resolution (Kavanagh et al., 2024). Physical attractiveness and facial expressivity are, however, intimately connected as expressive individuals can be perceived as more attractive (Golle et al., 2014), and, in turn, attractive individuals can be perceived as more facially expressive (Rollings et al., 2024). Here we used still images to control for the effect of facial expressivity on attractiveness perceptions as literature to date has demonstrated that facial expressivity can confound attractiveness judgements (Obwegeser et al., 2024), with both type of expressivity (e.g. Ho et al., 2018; Tracy & Beall, 2011) and intensity of expressive behaviours (e.g. Golle et al., 2014) impacting perceived facial attractiveness. While there are reports that facial attractiveness may vary between static and dynamic stimuli (Satchell, 2019), we utilised static images in the current study in order to more accurately tease apart the effect of attractiveness from that of facial expressivity.

### Limitations and Future Directions

The current study, however, is not without its limitations. Importantly, the social interaction setting in our study was online, which is a technological innovation that in some ways does not reflect in-person social interaction. Thus, some nonverbal behaviours, important to in person contexts might have been missed. For example, perceived eye-contact, which could not be measured in the current online paradigm, can contribute to favourable perceptions of interaction partners (Hietanen et al., 2020). It is possible, therefore, that our findings might not generalise to an in-person context. Nevertheless, we feel confident that this online setting is an appropriate first step. First, this context allows us to isolate the face and minimise the influence of wider bodily or gestural behaviours. Second, we can record spontaneous facial movement in great detail from a frontal video angle. Finally, and importantly, online interactions are now commonplace (Lieberman & Schroeder, 2020) and online facial expressivity correlates highly with in-person expressivity and similarly contributes to the outcomes of the social interaction (Rollings et al., 2024). We invite future research to build on our findings and consider cross-modal communication signals and their impact on social positioning during group formation.

Some other methodological decisions are worth mentioning in light of possible limitations and future directions. In the current study data on ethnicity and cultural background was not recorded, however, cultural differences could play a role in how facial behaviours are used (McDuff et al., 2017) or interpreted (Tsai et al., 2019). As a next step, a cross-cultural comparison could shed light into how our findings translate among different cultural backgrounds. Further, a longitudinal approach could be used to indicate whether facial expressivity influences group-level outcomes over time. This seems plausible, as initial judgements of others have been found to be predictive of long-term friendship outcomes (Human et al., 2013) Moreover, experimental and longitudinal studies could also be used to test the causal relationship between FE and popularity as the current study’s correlational nature cannot determine directionality. Finally, we provide future studies with some considerations for improved measurement of some of the studied variables. Namely, rephrasing the behavioural intention item used to capture participants’ willingness to continue interaction past the study’s end could be done to better reflect genuine interest, as the current wording of this item might have been confusing to participants due to the lack of actual opportunity for them to interact with each other again. Similarly, researchers are encouraged to use multi-item measures of person-perception to improve reliability and reduce measurement error. These adjustments could strengthen the interpretability and robustness of findings in this context.

To conclude, the current study is the first to consider how facial expressivity impacts social popularity during naturalistic social interaction in groups. Our findings indicate that more expressive individuals occupy more central roles within the group and are therefore more socially popular. These results support the hypothesis that facial expression functions to facilitate and maintain social connections with others.

## Supplementary Information

Below is the link to the electronic supplementary material.

Supplementary Material 1

## Data Availability

The complete study processed data, and R are available on the Open Science Framework: https://osf.io/kgz9v/overview?%20view_only=342a30dbc7bc44489e5dd0693fa524da.
