# Supplementary material for: Facially Expressive People are More Popular in Newly Formed Groups: A Social Network Analysis
Source: J Nonverbal Behav. 2026 Apr 27;50(2):233–53. doi: 10.1007/s10919-026-00514-6 (PMC13253780; doi:10.1007/s10919-026-00514-6)
Supplement: Supplementary file 1 — Supplementary Material 1 [file 10919_2026_514_MOESM1_ESM.docx]

Supplementary Materials

Method

***Observer-rated items: inter-rater agreement.***

We computed inter-rater agreement using a random-effects intraclass correlation coefficient [ICC(1,k); Shrout & Fleiss, 1979]. Because each image was rated by a partially overlapping subset of raters (M ≈ 23.8 raters per target), ICC(1,k) provides an estimate of the reliability of the mean rating for each target. Inter-rater agreement was good to excellent across all constructs. Attractiveness showed the highest reliability (ICC = .84), followed by warmth/competence (ICC = .79), cooperativeness (ICC = .77), prestige (ICC = .77), trustworthiness (ICC = .75), liking (ICC = .74), engagement (ICC = .73), and dominance (ICC = .72).

***Expressivity Controls.***

To control for the fact that participants’ expressivity might be affected by how much time they spent talking during the interaction, we gathered some additional information. Specifically, at the beginning of the call, participants were asked to say a single sentence about themselves (as an ice breaker; participants were instructed not to disclose any identifying information about themselves). We used this portion of the video to code two additional measures of expressivity: Expressivity during Greeting (expressivity during a similar amount of talking per subject) and Expressivity during Listening (the score here was the average expressivity during listening to all other team members). Both of these measures were based on FACS coding via the iMotions software and reflected the average of the transformed Z-score distributions of each FE measure. Only a sub-section of videos were reliably coded by iMotions here, resulting in 224 observations of Greeting condition and 249 observations of Listening condition.

**Time Talking.**

Talking Time was measured as the percentage of time each participant spent talking in relation to the overall call duration, calculated using the equation bellow:

Percentage talking=time talking/total time ×100

Results

**Principal Component Analysis: Expressivity**

To examine whether the six expressivity measures reflected a single underlying dimension, we conducted a principal component analysis (PCA) and exploratory factor analysis (EFA). Prior to analysis, sampling adequacy was assessed. The Kaiser–Meyer–Olkin (KMO) measure indicated acceptable adequacy (KMO = .77), and Bartlett’s test of sphericity was significant, χ²(15) = 680.85, p < .001, confirming that the correlation matrix was appropriate for factor extraction.

A PCA using standardised variables revealed that the first component had an eigenvalue of 3.21 and accounted for 53.5% of the total variance, with all subsequent components explaining substantially smaller proportions (17.8%, 12.0%, 8.7%, 5.1%, and 2.8%). A scree plot and parallel analysis both supported the retention of a single component (see Fig. 1 and 2. respectively). Loadings on the first principal component (see Table 1) were uniformly positive and ranged from .28 to .50, suggesting a single dominant expressivity dimension. Taken together, these results strongly support a unidimensional model. A single expressivity score was therefore computed by averaging the transformed z-score distributions of each FE measure.

**Figure 1.**

*Scree Plot with 5 components*


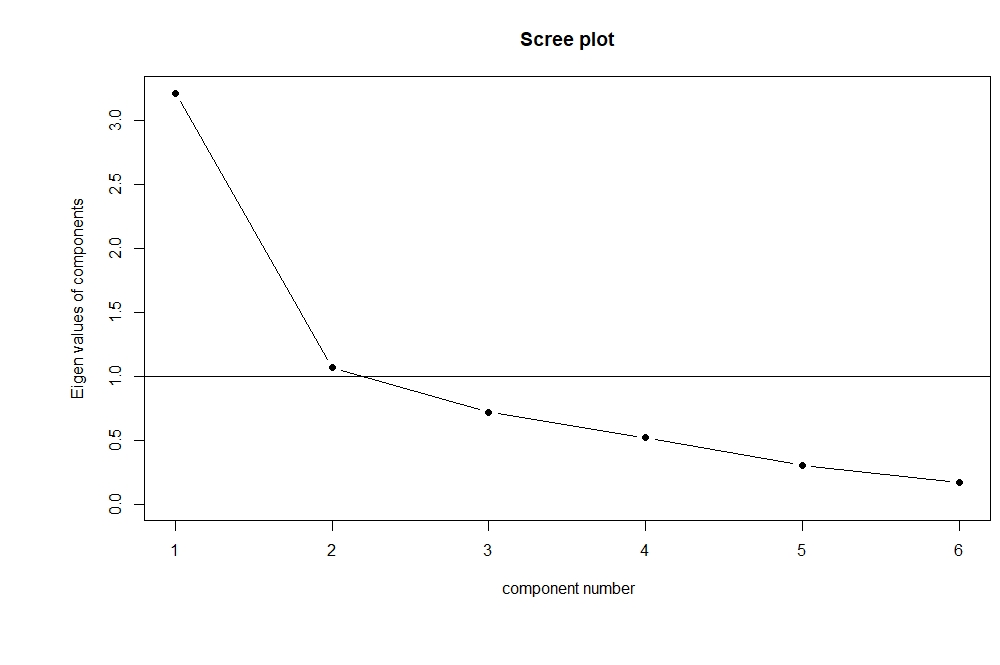


**Figure 2.**

*Parallel Analysis Plot*


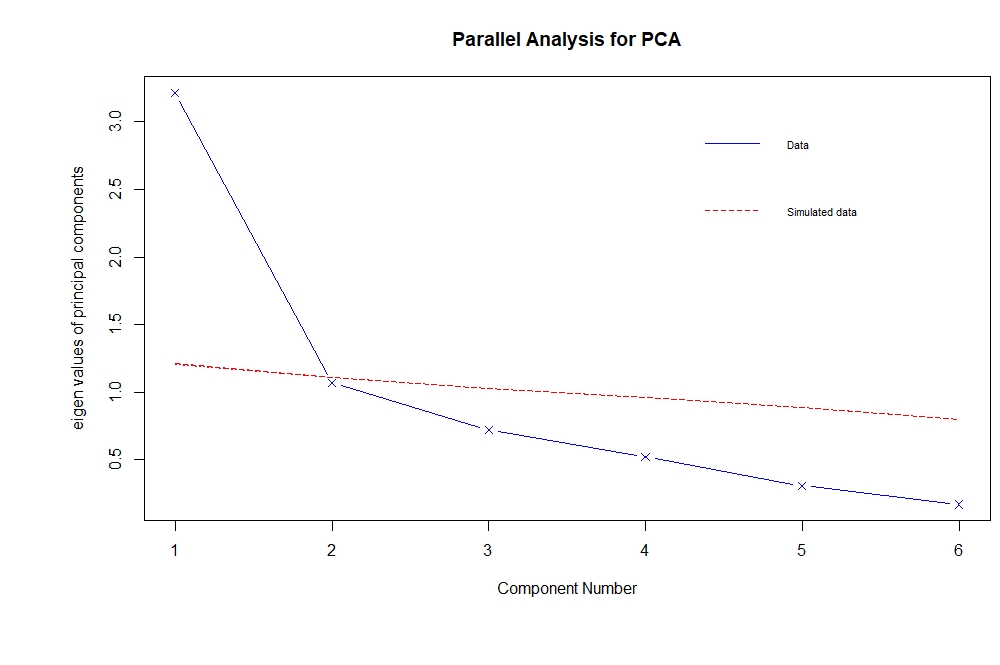


**Table 1.**
*Principal component loadings for the six expressivity measures.*

| **Variable** | **PC1** | **PC2** | **PC3** | **PC4** | **PC5** | **PC6** |
| --- | --- | --- | --- | --- | --- | --- |
| Rate | .48 | –.37 | .04 | .02 | –.37 | –.71 |
| Duration | .41 | –.47 | –.15 | .38 | .64 | .19 |
| Repertoire | .41 | .30 | .30 | –.66 | .45 | –.11 |
| Combination Repertoire | .50 | –.14 | –.03 | –.22 | –.50 | .66 |
| Diversity Score | .32 | .52 | .49 | .61 | –.08 | .03 |
| Corrected Repertoire | .28 | .51 | –.80 | .06 | .00 | –.12 |

**Expressivity Measures**

**Figure 2.**

*Pearson correlations between the three Expressivity measures*


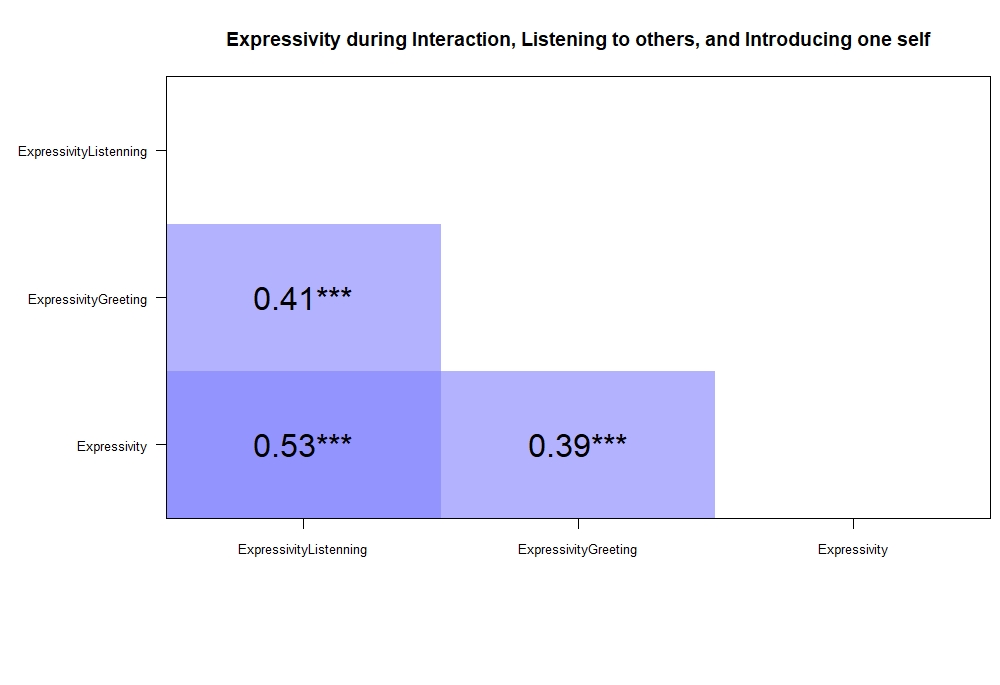


A linear mixed model was constructed with the *lmer* function from the *lmerTest* package and included Expressivity (during Listening) and Group Size as fixed effects, and the Group Number and the Participant that was Talking as random effects. The outcome variable was the Liking score received. The model revealed that facial expressivity during listening significantly predicted liking scores received, β=1.96, SE=0.86, t (572.73) =2.28, p=0.023. Group Size was not a significant predictor, β=-2.20, SE=2.71, t (78.80) =-.081, p=0.42. These findings suggest that people who display higher levels of facial expressivity during listening, are more favourably perceived by their group members.

**Degree Centrality**

To examine whether group size influenced degree centrality, we compared a linear mixed-effects model including a random intercept for Group with and without Group Size as a fixed effect. Including Group Size did not significantly improve model fit, Δχ²(1) = 1.56, p = .21, suggesting that group size did not account for additional variance in degree centrality beyond that captured by the random effect of Group. The models also showed negligible changes in information criteria (AIC = -537.99 vs. -537.54; BIC = -520.30 vs. -516.32), further indicating that including Group Size did not substantially improve model fit.

**Table 2.**

*Third-Party Perception Variables and Expressivity as predictors for degree centrality*

| Predictor | β | SE | df | t | p |
| --- | --- | --- | --- | --- | --- |
| Expressivity | 0.026 | 0.007 | 187.600 | 3.855 | < .001 |
| Perceived Attractiveness | -0.000 | 0.001 | 184.600 | -0.542 | .589 |
| Perceived Warmth | -0.001 | 0.002 | 185.500 | -0.451 | .653 |
| Perceived Competence | 0.001 | 0.001 | 187.600 | 0.958 | .339 |
| Perceived BI | 0.002 | 0.002 | 185.500 | 1.214 | .226 |
| Perceived Trustworthiness | 0.000 | 0.002 | 188.400 | 0.153 | .878 |
| Perceived Cooperation | -0.001 | 0.002 | 188.800 | -0.502 | .616 |

*Note: Linear Mixed-effects model with Group as a random intercept*

**Warmth and Competence Items Comparison**

To justify the aggregation of items and clarify the consistency of responses we compared items of warmth and competence and explored their distributions (see Table 3 and Figure 3 below).

**Table 3.**

*Variability in Warmth and Competence Items’ responses*

|  | Mean | SD |
| --- | --- | --- |
| Friendly | 82.4 | 14.9 |
| Positive | 81.5 | 15.1 |
| Likable | 81.3 | 15.8 |
| Competent | 80.5 | 15.6 |
| Capable | 80.8 | 15.6 |
| Able | 80.5 | 16.0 |

**Figure 3.**

*Pearson Correlations between all warmth (friendly, positive, likable) and competence (competent, capable, able) measures*


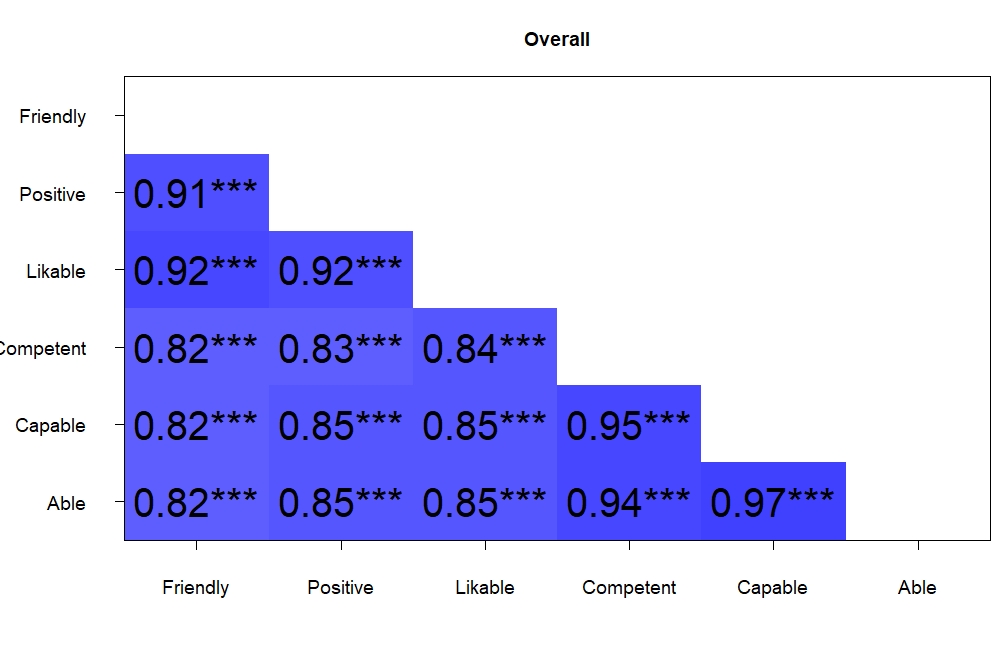


**Table 4.**

*Full models with Age and Gender as predictors*

| Outcome | Predictors | β | SE | df | t | p |
| --- | --- | --- | --- | --- | --- | --- |
| Degree Centrality | Expressivity | 0.023 | 0.006 | 190.54 | 3.56 | <0.001 |
|  | Attractiveness | 0.0003 | 0.0004 | 192.28 | 0.75 | .451 |
|  | Gender | -0.011 | 0.010 | 189.14 | -1.16 | .248 |
|  | Age | 0.004 | 0.001 | 190.20 | 2.93 | 0.004 |
| Warmth Perceptions | Expressivity | 3.907 | 1.0344 | 226.03 | 3.78 | <0.001 |
|  | Attractiveness | 0.173 | 0.070 | 231.46 | 2.47 | 0.014 |
|  | Gender | 1.099 | 1.571 | 221.90 | 0.69 | 0.485 |
|  | Age | -0.018 | 0.225 | 226.57 | -0.08 | 0.935 |
| Competence Perceptions | Expressivity | 1.889 | 0.971 | 221.72 | 1.95 | 0.053 |
|  | Attractiveness | 0.177 | 0.066 | 226.54 | 2.68 | 0.008 |
|  | Gender | 3.479 | 1.474 | 217.69 | 2.36 | 0.019 |
|  | Age | 0.144 | 0.211 | 221.14 | 0.68 | 0.494 |
| Cooperation Perceptions | Expressivity | 2.835 | 1.083 | 224.31 | 2.62 | 0.009 |
|  | Attractiveness | 0.146 | 0.073 | 229.29 | 1.98 | 0.048 |
|  | Gender | 0.778 | 1.645 | 220.15 | 0.47 | 0.637 |
|  | Age | 0.216 | 0.235 | 223.86 | 0.92 | 0.361 |
| Trustworthiness Perceptions | Expressivity | 0.739 | 1.334 | 222.774 | 0.55 | 0.580 |
|  | Attractiveness | 0.182 | 0.091 | 227.754 | 2.01 | 0.046 |
|  | Gender | -2.469 | 2.026 | 218.617 | -1.22 | 0.224 |
|  | Age | 0.291 | 222.251 | 1.518 | 0.13 | 0.291 |
| Behavioural Intentions | Expressivity | 1.259 | 1.720 | 205.038 | 0.73 | 0.465 |
|  | Attractiveness | 0.058 | 0.117 | 208.453 | 0.49 | 0.620 |
|  | Gender | -3.049 | 2.604 | 202.074 | -1.17 | 0.243 |
|  | Age | 0.374 | 204.062 | 0.250 | 0.81 | 0.374 |
| Cooperative Behaviours | Expressivity | -0.257 | 0.276 | 247.761 | -0.93 | 0.352 |
|  | Attractiveness | 0.001 | 0.019 | 247.481 | 0.07 | 0.946 |
|  | Gender | -0.417 | 0.423 | 246.448 | -0.99 | 0.325 |
|  | Age | 0.086 | 0.060 | 247.975 | 1.42 | 0.156 |
| Trusting Behaviours | Expressivity | 0.625 | 0.294 | 244.808 | 2.13 | 0.034 |
|  | Attractiveness | 0.015 | 0.020 | 247.428 | 0.78 | 0.437 |
|  | Gender | 0.205 | 0.448 | 241.927 | 0.46 | 0.649 |
|  | Age | 0.136 | 0.064 | 245.432 | 2.12 | 0.035 |
| Trustworthiness | Expressivity | 1.639 | 1.767 | 247.962 | 0.93 | 0.355 |
|  | Attractiveness | -0.119 | 0.119 | 246.035 | -1.00 | 0.318 |
|  | Gender | -0.365 | 2.707 | 247.706 | -0.13 | 0.893 |
|  | Age | 0.393 | 0.384 | 247.659 | 1.02 | 0.308 |
| Trusted Behaviours | Expressivity | 0.045 | 0.143 | 195.392 | 0.32 | 0.753 |
|  | Attractiveness | 0.013 | 0.010 | 197.594 | 1.37 | 0.173 |
|  | Gender | -0.040 | 0.216 | 193.416 | -0.18 | 0.854 |
|  | Age | -0.056 | 0.031 | 194.541 | -1.82 | 0.070 |

**Figure 4.**

*Correlation matrix between all measured variables*


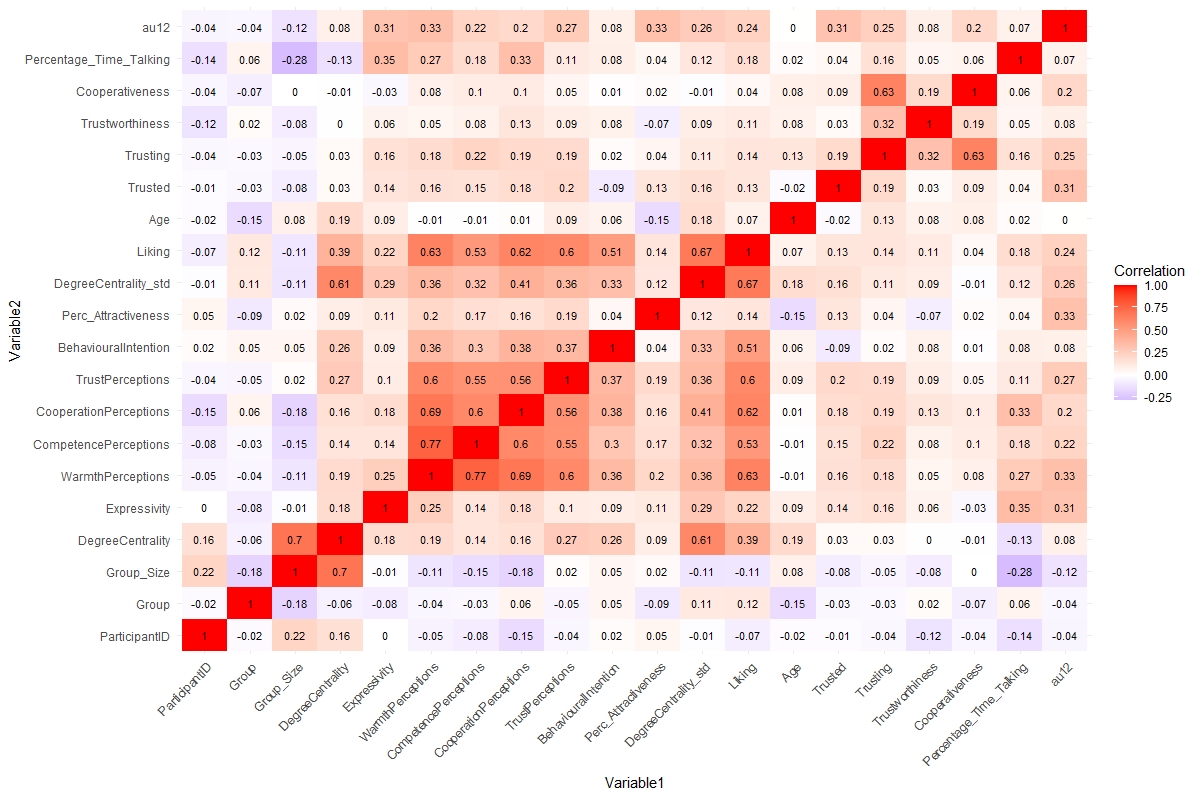


**Measures**

All measures are to be answered using a scale from 0 ( = not at all) to 100 ( = extremely).

1. Liking

How much do you personally like [Participant X]?

1. Warmth and Competence Perceptions*

To what extent do the following words describe [Participant X] in your opinion?

Thoughtful (warmth)

Friendly(warmth)

Positive (warmth)

Likable (warmth)

Competent (competence)

Capable (competence)

Able (competence)

* The questions for warmth and competence are adapted to the current study from Williams and Bartlett (2015).

1. Trustworthiness Perception

In your opinion, how trustworthy is [Participant X] that you interacted with in this study?

1. Cooperativeness Perception

In your opinion, how cooperative is [Participant X] that you interacted with in this study?

1. Behavioural Intentions (a.k.a. Behavioural Approach Tendencies)

How likely are you to engage with [Participant X] in further conversation outside of this study?

1. Attractiveness

How attractive do you find this participant?

Williams, L. A., & Bartlett, M. Y. (2015). Warm Thanks: Gratitude Expression Facilitates Social Affiliation in New Relationships via Perceived Warmth. *Emotion, 15*, 1–5. <http://dx.doi.org/10.1037/emo0000017>
